# Supplementary material for: A review of measures of women’s empowerment and related gender constructs in family planning and maternal health program evaluations in low- and middle-income countries
Source: BMC Pregnancy Childbirth. 2017 Nov 8;17(Suppl 2):342. doi: 10.1186/s12884-017-1500-8 (PMC5688455; doi:10.1186/s12884-017-1500-8)
Supplement: Supplementary file 1 — Evaluation papers included in systematic review. (DOCX 27 kb) [file 12884_2017_1500_MOESM1_ESM.docx]

**Additional File 1. Evaluation Papers Included in Systematic Review**

| **Transformative Interventions** | | | | | | |
| --- | --- | --- | --- | --- | --- | --- |
| **Study and country** | **Intervention components** | **Sample and design** | **Measured women’s empowerment or gender construct** | **Reported FP or MH outcome(s)** | **Results** | **Gender-effect for FP/MH outcome observed?** |
| Daniel et al. 2008 [14]  India | Behavior change communication (BCC) around RH and FP services  Improve access to RH services  Change social norms related to early childbearing | Male and female adolescents (18-24 yrs; married)  Quasi-experimental | None measured | Contraceptive use  Demand for contraception | AOR=3.84 (p<.001)^¥^  AOR=1.49 (p<.01)^¥^ | N/A: Gender measures not collected |
| Erulkar et al. 2009 [15]  Ethiopia | Mentorship  Non-formal education  Livelihoods training  Change social norms related to child marriage | Girls (10-19 yrs; unmarried)  Quasi-experimental | Girls’ access to safe spaces  Formal education/literacy | Child marriage  Ever-use of contraceptives among sexually experienced | In project area at endline, adjusted hazards ratio (AHR) for 10-14 yr older =0.009 (p<.001); AHR for 15-19 yrs olds=2.41 (p<.001)  AOR=2.88 (p<.01)^£^ | Yes, indirect for social networks, which increased in intervention areas. FP outcome improved in intervention sites, but social network not included as a mediator between program participation and FP outcome.  Yes, direct for education, which increased in intervention sites and included as a mediator between program participation and FP outcome. |
| Exener et al. 2009 [16]  Nigeria | Group health education | Males (from teens to >50 years old; married and unmarried) | Decision-making power in SRH; and social life  SRH control | Condom use at last intercourse  Number of unprotected vaginal sex occasions | AOR=4.10 (p<.001)^Ω^  AOR=0.2 (p<.05)^Ω^ | Yes, direct. |
| Lundgren et al. 2005 [17]  El Salvador | BCC around RH and FP services  Community-based provision of condoms  Referrals to other methods | Men and women (15-49 yrs; married)  Cross-sectional | Communication around SRH matters  Gender equitable attitudes (of woman or partner) regarding sexual and reproductive health (SRH) | Contraceptive use | OR=1.68 (p<0.001)^t^ | N/A: Association between gender measures and FP outcome not examined |
| Shattuck et al. 2011 [18]  Malawi | Provision of information on FP services  Strengthening communication/negotiation skills among couples  Positively influence attitudes towards FP and motivation to adapt FP | Men (≥18 years old; married or in partnerships)  Randomized control trial (RCT) | Gender equitable attitudes  Communication around SRH matters | Contraceptive use | AOR=2.5 (p<0.05)^Ω^ | Yes, direct for frequency of communication, which was significant predictor of contraceptive use |
| Venguer et al. 2007 [19]  Mexico | Provision of life skills curricula, including communication and negotiation skills to improve nutrition, hygiene and sexuality and RH | Women and girls (12-30 yrs; married and unmarried)  Quasi-experimental | Confidence/self-esteem | Contraceptive use  Pap Smear | Increased contraceptive Log odds =0 .085(p<.05) ^¥^  Had pap smear test Log odds = 0.551 (p<.05) ^¥^ | N/A: Gender was not included as a mediator between intervention arm and FP/MH outcomes |
| **Accommodating interventions** | | | | | | |
| **Study and Country** | **Type of intervention** | **Sample and Design** | **Measured women’s empowerment or gender construct** | **Reported FP or MH Outcome(s)** | **Results** | **Gender-effect for FP/MH outcome observed?** |
| Aubel et al. 2004 [20]  Senegal | BCC around workload of pregnant women | Grandmothers; mothers (15-29 yrs; married)  Quasi-experimental; Focus group discussions | None –construct emerged from qualitative study | Workload of pregnant women  Food intake of pregnant women | Decreased workload of pregnant women (91% vs 34%)^t^  Increased food intake of pregnant women (90% vs 35%)^t^ | N/A: Gender not measured quantitatively |
| Feldman et al. 2009 [21]  Mexico | Positively influence attitudes towards FP and motivation to adapt FP | Women (15-29 yrs; married)  RCT | Domestic and financial decision-making power | Use of modern contraceptives | Log odds = 0.16 (p<0.05)^α^ | No. Analysis included autonomy as mediator between program participation and contraceptive use, but no association found. |
| Mahamed et al. 2012 [22]  Iran | Health education around FP | Women (20-28 yrs; newly married)  Quasi-experimental | Gender equitable attitudes (of woman or partner) regarding sexual and reproductive health (SRH); and domestic matters | Knowledge and attitudes around FP | Improvement in knowledge (p<0.001) and attitudes (p<0.001)^£^ | N/A: Gender attitude was embedded in the FP outcome; FP outcome did not measure intentions or use. |
| Midhet etl al. 2010 [23]  Pakistan | BCC for empowerment for women and husbands  Training of birth attendants in obstetric danger signs  Structural (providing telecommunication and transport services for emergency obstetric care) | Pregnant women (married; age not specified)  RCT | Male engagement in health | Contraceptive use  Delivery in a hospital | None  None | N/A: Gender variable was not included as a mediator between intervention arm and the FP outcome |
| Mullany et al. 2009 [29]  Nepal | Health education on FP and MH | Women (> 18 years old; married)  RCT | None measured | Maternal and reproductive knowledge (based on 7 questions) | Improved knowledge among in 5 out of 7 questions; borderline statistically significant (p<0.05)^£^ | N/A: Gender not measured quantitatively. |
| Mushi et al. 2010 [24]  Tanzania | Community mobilization around safe motherhood  Training "safe motherhood promoters" | Pregnant women and mothers; and their husbands (19-53 yrs; married)  Longitudinal; Qualitative interviews | None –construct emerged from qualitative study | Deliveries with skilled attendant | Increased use of skilled attendant (51.4% at endline vs 34.1% at baseline), compared to 36% at regional level at both time points (p<0.05) | N/A: Gender not measured quantitatively |
| Nasreen et al. 2012 [25]  Bangladesh | BCC around FP and MH  Engagement of community leaders (Imams and village doctors) | Males (26-40 yrs; married)  Quasi-experimental | Male engagement in health | Men's knowledge of FP and awareness of their wives' experience of abortion  Men's knowledge of MH and awareness of of their wives' ANC use | None  Increased knowledge of saving money for birth preparedness (p<0.001); Increased knowledge of determining attendant at delivery (p<0.001) and buying delivery kit (p<0.001)^Ω^ | N/A: Gender measures were same as FP outcome |
| Sahip and Turan 2007 [26]  Turkey | Group health education | Men (late 20s – early 30s; married; expectant fathers); Wives of the men  Quasi-experimental | Male engagement in domestic matters; and in 3health | Contraceptive use  Use of antenatal and postnatal care | None  None | N/A: Gender variables were not included as mediators between intervention group and FP and MH DVs |
| Sathar et al. 2005 [27]  Pakistan | Training health care providers on a client-centered approach to care | Clinics (facility infrastructure, equipment and staff); Community based workers  Quasi-experimental | Service provider-client interaction | Behavior of service providers towards female clients | Result for overall program not significant.  Difference-in-difference (DID) coefficient for Assessment = 0.35 (p<.05).  DID coefficient for Assessment = .30 (p.05); DID coeffcient for Help=.41 (p<.05) | Yes, for some outcomes |
| Tang et al. 2009 [28]  China | Provision of information on FP and RH using web-based strategy | Service providers: village doctors, FP workers, women's cadres, teachers  RCT | Attitudes around women’s health | Contraceptive knowledge  Provider clinical skills & knowledge of antenatal, delivery and postnatal care | Improvement in multiple indicators of contraceptive knowledge^£^  Improvement in multiple indicators of provider clinical skills & knowledge of antenatal, delivery and postnatal care^£^ | N/A: Gender was not included as a mediator between intervention arm and FP/MH outcomes |

^¥^ Interaction term of time*study group

^£^ Endline vs. baseline in intervention group

^Ω^ Intervention vs comparison group at endline

^t^ Endline vs baseline of separate cross-sections in intervention group

^α^ Midline vs baseline in intervention group

N/A indicates that the study did not examine the relationship between gender and the FP or MH outcome variable
